# The Father’s Part: A Pilot Evaluation of a Father-Centered Family Intervention Group in Child and Adolescent Psychiatry

**DOI:** 10.3390/bs14010013

**Published:** 2023-12-22

**Authors:** Stefan Mestermann, Jonas Markus Kleinöder, Marie Arndt, Josef Krämer, Anna Eichler, Oliver Kratz

**Affiliations:** Department of Child and Adolescent Mental Health, University Hospital Erlangen, Friedrich-Alexander-Universität Erlangen-Nürnberg (FAU), 91054 Erlangen, Germany; jonas.kleinoeder@uk-erlangen.de (J.M.K.); oliver.kratz@uk-erlangen.de (O.K.)

**Keywords:** parental behavior, parenting stress, child development, parent–child-relation, parent-centered interventions

## Abstract

Changes in parental roles have renewed the focus on a father’s involvement in an offspring’s psychological development. However, fathers are still under-represented in family research. There are only a few structured father-centered intervention programs in child and adolescent psychiatry. In a German population sample, a pilot father-centered family intervention program with n = 16 participants, conducted in person (n = 8) and online (n = 8), in a child and adolescent psychiatry inpatient/day clinic setting was evaluated by comparing paternal stress, PSE, and child-rated paternal competence in a pre–post design. Participating fathers showed significant decreases in child-related parenting stress (presence: *p* = 0.042, online: *p* = 0.047) and significant increases in PSE (*p* = 0.006/0.012). Parent-related stress and child-rated paternal competence were unaffected (*p* = 0.108/0.171; *p* = 0.167/0.101), while small-to-medium effect size measures pointed in the direction of our hypothesis (*d* = 0.48/0.36; *d* = 0.37/0.50). Participant satisfaction was higher in person than online (*p* = 0.008). As social and biological fathers have important influences on child and adolescent well-being and development, they should be included more frequently in prevention and intervention programs. Fathers seem to benefit from gender-specific intervention programs with regard to stress reduction, as well as experiencing competence- and PSE-increasing effects.

## 1. Introduction

The concept of the family and role distributions have undergone several changes over the last few decades [1]. Family diversity allows mothers, fathers, and other caregivers to share the responsibility of education, childcare, and support. However, they continue to be faced with the increased childcare demands of modern civilization, causing parental distress [2]. Parental stress, subjective parental self-efficacy (PSE), and competence are among the established determinants of offspring mental health [3,4].

Formerly, hypotheses on conservative family models—when childcare was mainly a maternal task—focused on a deficit approach, with the aim of assimilating fathers and mothers [5,6]. Current family research examines the specific contributions of mothers, fathers, and other caregivers to child and adolescent development [7]. New family models, including sexual-minority-parent families, are increasing [8] and not all of them may have father figures. The focus on social and biological fathers’ (both addressed by the term ‘fathers’ in this article) involvement in childcare and offspring psychological development has been renewed. By examining minors without any clinical symptoms, several studies have outlined the significant paternal influence on offsprings’ cognitive and emotional maturation, e.g., via functional father–child play [9] or engaged and sensitive attention [10]. Our latest work showed significant paternal influence, particularly regarding psychopathology and parenting style, on child and adolescent mental health [11]. Father support and empathy encourage pro-social behavior in adolescents and young adults [12]. Furthermore, fathers play an important role in families with mentally ill children; internalizing (e.g., emotional disorders, depression, anxiety) and—particularly for boys—externalizing disorders, like attention deficit hyperactivity disorder (ADHD), or conduct disorders, are associated with less positive paternal parenting behavior or involvement [13,14]. Challenges during the COVID-19 pandemic highlighted these connections [15].

Like mothers and other caregivers, fathers experience childcare demands. In particular, impaired father–child bonding, less time spent together, and frequent interactional conflicts increase the risk of paternal distress [16,17]. PSE (parents’ belief in their ability to successfully perform their parenting role [18]) correlates with functional (e.g., authoritative) parenting styles [19] and is associated with higher parental mental well-being [20]. Alternatively, low PSE correlates with higher child [21] and parent psychopathology, less involvement in parenting tasks, and partnership conflicts [22]. In this regard, there are fewer studies involving fathers than mothers. Among the predictors of paternal PSE are active participation in parenting [23], positive partnership quality, and low overall paternal distress [24]. Additionally, fathers’ PSE is postulated to increase paternal and child psychological well-being/development and paternal warmth in parenting [25], as well as lead to positive involvement in parenting tasks [26].

Parental competence is a less concretely defined construct. In family research, it mainly refers to parenting practices [27], which—alongside sociocultural family factors—can have significant impacts on psychomotor, emotional, and cognitive child development [28]. Dimensions can be child- (empathy, support), context- (social/material well-being), parent- (knowledge of parenting, emotion regulation), and interaction-centered (e.g., consistency) [29]. This construct is less addressed in the literature, mostly because it is more difficult to quantify. Thus, parental competence is usually addressed via subjective assessments, mostly by mothers and/or children [30]. Several instruments use minors’ perception of the child–parent relationship and attachment as indicators. Father-specific data on perceived competence are lacking. The literature postulates bidirectional correlations with fathers’ stress levels. Paternal psychopathology and child demandingness are among the associated factors [11,31,32]. Further, paternal competence seems to directly correlate with a father’s involvement in parenting [33].

Despite these findings, pediatric and child/adolescent psychiatric treatments mainly focus on a mother’s involvement in diagnosis and therapy [34,35]. Work with and support for parents and other caregivers is, however, an essential part of psychological care for minors. Systemic approaches should enable families to change dysfunctional interaction patterns and other disorder-maintaining factors in a family setting [36]. Thus, parent-centered support and training programs in the psychological diagnosis and treatment of children and adolescents are of high importance, and their follow-up effects are well established [37]. For example, specific training concepts for the caregivers of children with autism spectrum disorders (ASDs) include ‘Applied Behavioral Analysis’ (ABA), ‘Pivotal Response Training’ (PRT), ‘Treatment and Education of Autistic and Related Communication-Handicapped Children’ (TEACCH), and ‘Behavioral Skills Training’ (BST) [38,39]. Conduct disorders and disruptive behavioral problems are addressed by interventions like the ‘Incredible Years Program’, ‘Triple P’, or ‘Parent Child Interaction Therapy’ (PCIT) [40,41]. Most of these manuals include both parents or do not specify the involved parents and other caregivers. There are not many exclusively father-centered structured parental training programs in child and adolescent psychiatry. However, some gender-specific parent interventions exist, and also some specifically focusing on fathers; for example, the English manual ‘Coaching Our Acting-out Children: Heightening Essential Skills’ (COACHES) is specifically designed for fathers supporting the mental health treatments of children with ADHD. The eight-week training program combines the use of cognitive–behavioral strategies with recreational (mainly physical) activities (e.g., soccer drills and other games) for the development and training of fundamental positive parenting skills (e.g., homework support, reinforcement, timeouts) [6,42]. For young German patients and their families, there are no standardized and manualized father-centered intervention programs.

Most studies and manuals include in person parent-centered interventions with face-to-face instructions, parent–child tutorials, and the immediate implementation of parenting skills in ‘live’ situations. Most recently, not least because of the COVID-19 pandemic and the related lockdowns, the possible benefits of online-based parent interventions are a subject of research. Current publications postulate a comparable effectiveness of presence and telehealth groups, e.g., for ASD-specific programs [39], (prevention of) substance use, and for the improvement of family functioning [43]. The information and exchange platform ‘New Fathers Network’, which is in the English language and provides opportunities to gain knowledge about infants’ needs, development, and health, was found to increase fathers’ postnatal self-efficacy and contentedness [44].

Study aims: Although family research is increasingly focusing on parent-specific influences on child development and mental health, fathers are still underrepresented in the psychological treatment processes of minors [34], and children and adolescents may significantly benefit from a paternal involvement [6,45]. There are only few structured father-centered interventions in child and adolescent psychiatry, and none in the German language. There is a gap in the literature for reviewing fathers’ participation in child and adolescent psychiatric treatments and its acceptance. Paternal support and influences on family systems as well as the outcome of minors’ mental diseases have not yet been sufficiently clarified. The aim of this pilot study was to evaluate a father-centered family intervention program and support group concept from fathers’ and children’s perspectives in a child and adolescent psychiatry inpatient/day clinic setting in Germany. (1) Pre/post intervention measures of fathers’ parenting stress, PSE, and child-rated subjective paternal competence were assessed and compared. Additionally, we (2) focused on the intercorrelations and associations of the outcomes with child characteristics and sociodemographic factors. (3) Participant satisfaction was described.

## 2. Materials and Methods

### 2.1. Intervention and Study Design

**Figure 1:**
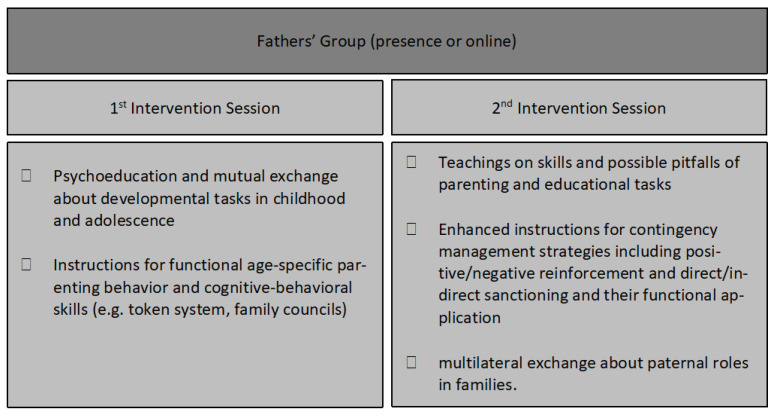
Content of the first and second intervention sessions of the father-group, also based on German therapy manual for parents of mentally ill children ‘Eltern stark machen’ (Eng.: ‘Strengthen Parents’) [46].

The father-group was developed as a structured father-centered gender-specific family intervention program at our Department of Child and Adolescent Mental Health, University Hospital Erlangen, Germany, to reduce paternal stress and to increase paternal parenting self-efficacy and competence. In the present study, the father-group study was conducted in two cohorts: group 1, in person (starting October 2020), group 2, online (starting February 2021). Both cohorts underwent two intervention sessions (of 90 min) with a three-week interval. The interventions were led by two male child and adolescent psychiatry professionals. In a group therapy setting for relatives, fathers received therapeutic and pedagogical instructions for a daily routine for dealing with their mentally ill children, with specific emphasis on educational/parenting difficulties and related differences across developmental periods. Subjective paternal tasks and challenges in parenting and child development were particularly identified and addressed, including possible solution strategies. Paternal roles and parenting styles were reflected in systemic family contexts, and cognitive–behavioral/contingency management strategies for parenting, like token systems and family councils, were conveyed. A large focus of the group intervention was on mutual exchange between the participants. The intervention design was partially based on the German therapy manual for parents of mentally ill children ‘Eltern stark machen’ (Eng.: ‘Strengthen Parents’) [46]. The detailed content of both intervention sessions is summarized in Figure 1.

**Figure 2:**
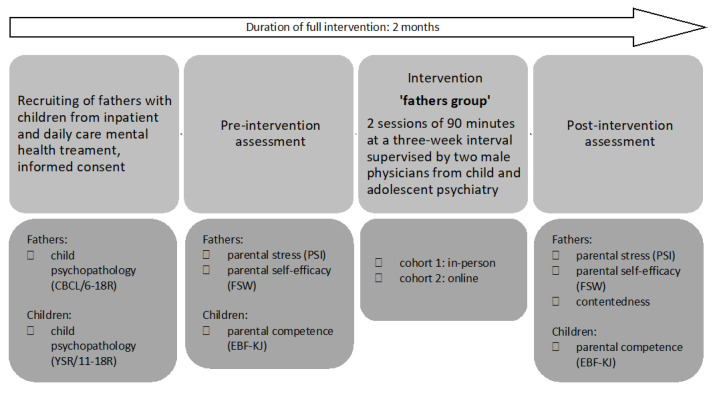
Study design. CBCL/6-18R: Revised Child Behavior Check List [47], YSR/11-18R: Revised Youth-self-report [48], PSI: ‘Parenting Stress Index’ [49]; FSW: German questionnaire ‘Fragebogen zur Selbstwirksamkeit in der Erziehung’ [50], EBF-KJ: German questionnaire ‘Elternbildfragebogen für Kinder und Jugendliche’ [51].

Father–child dyads were recruited from the inpatient and daily care units of our Department of Child and Adolescent Mental Health, University Hospital Erlangen, Germany. Informed consent (adults) or assent (minors) was obtained from all participants involved. The study was approved by the Local Ethics Committee of the University Hospital, Erlangen (380_20 B) and was conducted in accordance with the Declaration of Helsinki. Inclusion criteria were current treatment of the father’s child at our Department and provision of informed consent and assent, as well as participation in all assessment and intervention sessions. Participant dyads were excluded if regular attendance was not provided and if either father or child did not agree to the study participation. At the pre-intervention assessment (inpatient/day clinic admission), fathers and children answered standardized questionnaires regarding child psychopathology. In a pre-/post-test assessment, fathers self-reported subjective parental stress and PSE in a paper-and-pencil format. Likewise, children rated paternal competence levels via questionnaires. Figure 2 shows the detailed intervention and evaluation protocol.

### 2.2. Sample Characteristics

A total of n = 16 father–child dyads participated in the intervention, with n = 8 dyads per cohort (in person or online). The children’s ages ranged from 10 to 17 years. All fathers stated to be the children’s biological parents. Detailed demographic sample characteristics of fathers and adolescents are summarized in Table 1; there were no significant differences between the in-person vs. online format.

### 2.3. Data Acquisition

Child psychopathology was addressed using the German versions of the ‘Child Behavior Check List’ (CBCL/6-18R) (father-rating) [47] and ‘Youth Self-Report’ (child-rating) (YSR/11-18R) [48] in a pen-and-paper format. Both questionnaires use the subscales Aggressive Behavior, Anxious/Depressed, Attention Problems, Rule-Breaking Behavior, Somatic Complaints, Social Problems, Thought Problems, and Withdrawn/Depressed, which are summarized in three global scales: Internalizing Score, Externalizing Score, and Total Problems Score. T-values ≥ 60 indicate borderline significance, T ≥ 64 indicates significant clinical relevance. In the present study, children/adolescents (YSR) and fathers (CBCL) rated the questionnaires during clinic admission.

(Child- and parent-related) Paternal parenting stress was measured using a revised German version [52] of the ‘Parenting Stress Index’ (PSI) [49]. It assesses child-related (distractibility/hyperactivity, mood, acceptability, demandingness, adaptability) and parent-related dimensions (attachment, isolation, competence, depression, health, role restriction, spouse/partnership quality) of parenting stress. Ratings on a 5-point-Linkert-scale (1 = ‘not at all’ to 5 = ‘exactly’) conclude child- [20 (lowest) to 100 (highest)] and parent-related stress sum scores [28 (lowest) to 140 (highest)]. In the present study, fathers provided self-reports via PSI at pre- and post-intervention assessment.

We quantified PSE using the German questionnaire ‘Fragebogen zur Selbstwirksamkeit in der Erziehung’ (FSW) [50]. It uses nine items (e.g., ‘It’s easy for me to be a loving father/mother’), which are rated on a 4-point Linkert scale (from 1 = not true to 4 = completely true). A raw sum score (9 to 36) represents subjective PSE lowest to highest. In our study design, fathers completed FSW before/after the group intervention.

Paternal competence was addressed via the German questionnaire ‘Elternbildfragebogen für Kinder und Jugendliche’ (EBF-KJ) [51], with nine dimensions (cohesion, identification, autonomy, conflict, punishment, rejection, emotional collection, over-protection, and help). T-values ≤ 35 indicate significantly low and, ≥65 indicates above average, parental competence. In the present study, children and adolescents completed EBF-KJ during pre- and post-intervention assessments.

Participant satisfaction with the group intervention was evaluated via an individually designed questionnaire. Fathers rated 10 items (e.g., ‘How do you rate overall quality of the intervention?’) on an interval scale following German school grades (1 = ‘very good to 6 = ‘insufficient’). A raw sum score was calculated with a 1 to 6 range.

### 2.4. Statistical Analyses

Data were analyzed using IBM^®^ SPSS^®^ Statistics (Version 26.0). The level of significance in all analyses was defined as *p* < 0.05 (two-tailed), and trend-significance as *p* < 0.10. Normal (Gaussian) distribution was evaluated via Shapiro–Wilk test; variance homogeneity was tested using the Levéne test. Descriptive group comparisons were conducted via paired *t*-tests or Chi-square tests. Pre-/post-intervention data were compared using paired *t*-tests or Wilcoxon signed-rank tests with effect size measured using Cohen’s d (respectively, z for Wilcoxon), interpreted as 0.1–0.3 (weak/small), 0.3–0.5 (moderate/medium), and >0.5 (strong/large) [53]. In order to detect inter-outcome associations and outcome correlations with sociodemographic/child characteristics, Pearson’s (r_p_), Spearman’s (r_s_), or point-biserial (r_pb_) correlations were calculated depending on scale levels, with |r| ≥ 0.10 being considered low correlation, |r| ≥ 0.30 moderate, and |r| ≥ 0.50 strong/high correlation. Post-hoc, we computed the average effect size (d_M_) and the achieved power of the present study design, as well as the required sample size for significant effects in future studies [54].

## 3. Results

### 3.1. Child Psychopathology

There were no significant differences in child psychopathology ratings between the in-person and online cohort at hospital admission. Children showed clinically relevant internalizing or externalizing problems (Table 2).

### 3.2. Paternal Characteristics

Child-related parental stress was reduced significantly in both the in-person and online cohort between t1 and t2 (*p* = 0.042/0.047), with a medium effect size (d = 0.71/0.69; Table 3).

Parent-related parental stress dimensions did not differ significantly before/after the in-person (*p* = 0.108) and online (*p* = 0.171) interventions. The effect size showed small effects in a hypothesis-conforming manner (d = 0.48/0.36; Table 3).

PSE significantly increased between pre- and post-intervention in both cohorts (*p* = 0.006/0.012). Effect sizes were large (z/d = 0.57/1.02; Table 3).

**Figure 3:**
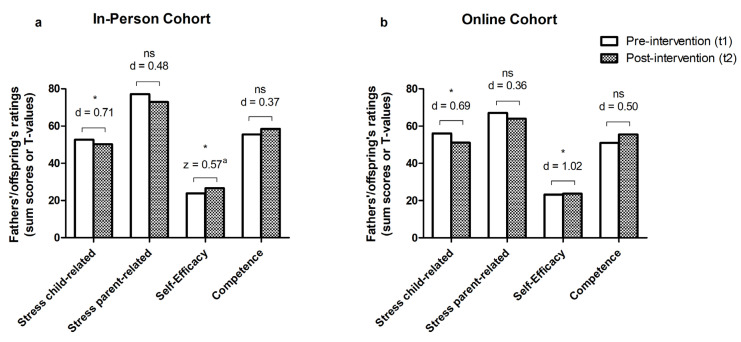
Pre-/post-intervention comparisons of father’s child- and parent-related stress, self-efficacy, and competence in the in-person (**a**) and online (**b**) group format. Stress child-/parent-related: PSI: Parenting Stress Inventory [49]; self-efficacy: FSW: German questionnaire ‘Fragebogen zur Selbstwirksamkeit in der Erziehung’ [50], competence: EBF-KJ: German questionnaire ‘Elternbildfragebogen für Kinder und Jugendliche’ [51], ns: not significant, * *p* < 0.05, |d|: Cohen’s d/*t*-test effect size [for parental self-efficacy in the in-person cohort, Wilcoxon signed-rank test was performed instead of *t*-test. ^a^ z for the effect size of Wilcoxon signed-rank test)].

Child ratings on paternal competence did not differ significantly before/after group intervention in the in-person (*p* = 0.167) or online (*p* = 0.101) cohort (Table 3, Figure 3). The effect size showed small to medium effects in a hypothesis-conform manner (d = 0.37/0.50).

### 3.3. Participant Satisfaction

Overall, satisfaction was very good to good. Fathers in the in-person group reported significantly higher contentedness with the intervention (M = 1.30, SD = 0.32) than participants in the online cohort (M = 2.10, SD = 0.53; *p* = 0.008).

## 4. Discussion

Family research is often lacking mother–father specification in developmental, educational, and mental health investigations, particularly as fathers are less involved in clinical mental health treatments of children and adolescents. Specifically within a clinic setting, fathers’ engagement is poor [6]. High maternal child-care, fathers’ working habits, and therapists’ selection bias are among previously postulated reasons [34,55]. These factors seem have an even larger impact on families with low social status [56,57]. Additionally, fathers in prior studies were less open to changes in parenting [58] and less accepting of family interventions [59]. Admitting the need for external help seems to be less pronounced among men [60]. Thus, it is important to validate caregiver-specific contributions in child mental health development and therapy [6]. The present study marks an important step toward father-inclusive therapeutic interventions in systemic child and adolescent psychiatry in Germany.

### 4.1. Father-Group Intervention Effects

Fathers participating in both cohorts reported a significant reduction in child-related parental stress at high effect size. This aligns with findings in the literature, in which effective distress reductions are seen following parent-interventions are described in healthy families [61]. Similar results are also seen during the treatment of externalizing disorders, like ADHD [62], conduct disorders [63], or ASD [64,65]. However, all adolescents in our study were reported to have internalizing mental diseases, like anxiety and depression. Although there are fewer structured intervention programs for these disorders, effectiveness is postulated as well [66]. The main topics in our intervention, developmental tasks in childhood and adolescence, functional age-specific parenting behavior, and cognitive-behavioral parenting skills, are likely to strengthen fathers’ self-confidence and make them feel ‘prepared’ for difficult situations (e.g., conflicts) at home. Additionally, the strong emphasis on mutual and therapeutic exchange may have led to an experience of acceptance and being understood. It has to be noted that, all children/adolescents of fathers included in our study received child and adolescent psychiatric treatment during the intervention, which limits the validity regarding isolated father-group effects. Inpatient/day care treatment may have additionally reduced paternal stress and/or increased PSE and competence as a result of child symptom reduction. For that purpose, we suggest further research including Treatment-As-Usual (TAU) control groups, to further investigate the specific benefits of our pilot father-centered intervention. However, fathers may benefit from the ‘self-help character’ of the intervention rather than just being instructed in one-on-one-instructions by therapists [67]. It may widen fathers’ knowledge about functional authoritative parenting strategies, and thereby diminish possible anticipatory strain at home.

In our study, father-related stress was reduced with a medium effect size, yet not significantly. Parent-related distress is considered to be a more constant/steadier variable [68]. Most notably, our cohorts were only of small sizes. This might be an explanation for why our limited-time 2-month intervention was not able to improve parent-related distress significantly, but a sufficient effect size was demonstrated. While father-related parental stress was not the main focus of the accompanying child/adolescent psychiatric treatment, a specific effect of the father-group is nonetheless assumable. Despite the need for confirmation of these findings in larger cohorts and by using TAU control groups, our intervention seemed to successfully reduce fathers’ parent-related stress. Further, only fathers (one caregiver from each dyad) were enrolled into the group, the mothers and other caregivers were not involved. This limits the effects on caregiver dyads. Some authors suggest a higher effectiveness of family interventions when both parents are involved [69,70,71]. Child/adolescent gender seems to be an additional co-variable. In our study, fathers’ parent-related stress correlated strongly with male child gender (see Supplementary Materials, Table S1). A possible reason might be that externalizing, and thus more distressing, disorders are more frequent among boys than girls [72].

Our cohorts reported increased paternal PSE during the post-intervention assessment with a high effect size. Despite the paucity of research into PSE, we were able to reproduce data from Salonen et al., who demonstrated a significant increase in fathers’ PSE after an online intervention. However, the age of the children was different (postpartum period) and there were less fathers than mothers involved (Δn/n ≈ 50%) [73]. A meta-analysis by Wittkowski et al. reported an overall enhanced PSE in healthy children’s parents after group-based interventions, but did not primarily focus on fathers [74]. PSE is a relevant bidirectional mediator of family functioning [4]. Fathers’ subjective PSE predicts warm father–child interactions [75]. Caregivers with low PSE tend to show overstrained impulsive reactions towards the child, resulting in harsh, aggressive, and dismissive parent behavior [4]. Functional experiences in education, mostly seen in authoritative parenting, directly enhance children’s and adolescent’s self-esteem [76]. Vice versa, these factors are known to enhance PSE as well, as demonstrated by the correlation between PSE and child-related stress in our study. Other family factors, like partnership quality, child’s siblings, and financial security, also play an important role [24,28,77]. Fathers of girls tend to be more attentive, emotional, and sensitive [78]. Father–daughter and mother–son interaction have a significant impact on child and adolescent mental health [11,79], which is supported by the strong correlation between fathers’ PSE and female-sex children in our in-person cohort (see Supplementary Materials, Table S1). Further, PSE is lowered by child behavioral problems [80]. We were able to demonstrate the effect the of the father-group on PSE. As increased PSE might positively influence various other parenting aspects (e.g., warm interaction, authoritative parenting style), focusing on paternal PSE as the target variable in parent-centered interventions is of high clinical relevance. Thus, a combination of multidisciplinary psychiatric treatment for children (addressing their mental diseases) and manualized interventions for parents and other caregivers (providing caregiver support) is likely to deliver the best possible outcomes for families.

The children and adolescents included in our study rated higher perceived paternal competence during the post-intervention assessment with a medium effect size. Yet, data were not significant, most likely as a result of the small cohorts. The literature describes inconsistent outcomes following parental competence programs [81]. Child perception of parental competence is postulated to be a stable construct, which is internalized during early childhood [82]. Furthermore, the children and adolescents were not part of the present father-group intervention. In addition to the low number of participants in our pilot intervention, the confined time gap of less than two months between the assessments, and the wide definition of parental competence, might be an explanation for non-significance. However, an increase in subjective paternal competence, as demonstrated by our data, is of clinical relevance, and thus a valid target for parent-centered interventions. In accordance with our findings (significantly strong negative correlation between perceived competence and child-related stress, see Supplementary Materials, Table S2), fathers’ parenting stress is known to impair subjective parental competence [31]. This reflects the high importance of functional child–parent relations and interactions; although the literature mainly focuses on parental self-reports regarding competence-ratings and child-/parent-related stress, differentiation between self-reports and third-party interviews is not always conducted.

### 4.2. Achieved Power and Required Sample Size: Post-Hoc Analyses

Post-hoc, we computed the achieved power of the present study design and the required sample size for significant effects in future studies. The average effect size of the present study’s outcome measures was d_M_ = 0.60 (see Table 3). The presented pilot study design achieved 31% power (1-β); meaning, the probability of the *t*-tests to find an effect, if there is an effect to be found, is 31%. Although caution is required in predicting study power [83], the sample size required for future studies to achieve statistically significant results at 80% power was calculated as n = 24 (*t*-test, pre-post without control group)/n = 15 per group (F-test, pre-post with control group).

### 4.3. Presence vs. Online Intervention

The effectiveness of the father-group in our study was comparable between the in person vs. online group format. The effect size for self-efficacy increase was higher in the online cohort, whereas the effect size for reduced child-related parenting stress was higher in the in-person group. The literature confirms the non-inferiority of telehealth parent interventions in different therapy settings, e.g., for ASD [39]. Online interventions for parents’ own mental health showed promising effectiveness, e.g., in parental depression and anxiety [84]. However, in our study, post-intervention satisfaction was higher in the in-person cohort. Yet, online interventions offer various beneficial characteristics: Online interventions are easier to access, which allows higher reachability and more participants in one intervention [84]. This results in more equal opportunities for low social status families [43]. In addition, higher cost-effectiveness and time-savings are possible. Some authors postulate that, specifically fathers are more likely to attend an online intervention [6]. Additionally, while interventions in person mostly take place in a clinical setting, online interventions are accessed from home, which may facilitate the transfer of the taught content to the domestic setting [43]. A limiting factor is that, for the final comparison of our in-person vs. online formats, repeated ANOVA analyses would have been necessary, which was not possible due to our small cohort sizes (n = 16). Future studies with higher numbers of participants are needed to conduct these calculations and confirm our results. Yet, as the satisfaction of both father-group cohorts was at least good, and as telehealth interventions offer various mentioned benefits, the online format remains a valid addition in the field of parent-centered interventions in child and adolescent psychiatry.

### 4.4. Limitations

This study has several limitations. We evaluated a pilot intervention program for fathers, which does not (yet) rely on manualized processes or validated outcome criteria. Both cohorts were of only small sample size. Further, we did not use control groups. The children/adolescents included received child and adolescent psychiatric treatment during the intervention, which limits the significance of isolated father-group effects. Our study design did not include post-intervention assessment of child/adolescent mental health. In future studies, a control group would have to be added, follow-up measures should ask for stability of effects, the sample size should be increased, and effects on child mental health outcomes should be examined to objectify effectiveness and standardize the father-group as a manualized father-centered parent intervention program.

## 5. Conclusions

Social and biological fathers have important influence on children’s well-being and development. Fathers seem to benefit from gender-specific interventions in the context of child and adolescent psychiatry: The father-group intervention led to reduced child- and parent-centered stress, increased paternal PSE, and higher child-rated paternal competence in the in-person and online format. In conclusion, for the clinical context, fathers should be included more frequently in child/adolescent mental disorder prevention and intervention programs. Father-focused treatment approaches can expand therapeutic possibilities in child mental health treatment and should be offered regularly as a module in psychotherapeutic diagnostic and therapeutic processes. Child and adolescent health specialists should keep in mind that paternal contribution can significantly improve mental disease diagnostics and therapy and that, where acceptable and possible, all (supporting) caregivers should be enabled to participate in treatment.

## Figures and Tables

**Table 1:** Demographic sample characteristics of the father-group participants.

		Cohort 1 (Presence, n = 8)	Cohort 2 (Online, n = 8)		
		M/N (SD/%)		t (df)/χ^2^ (df)	*p*
Fathers					
Age [y]		51.0 (5.3)	48.4 (7.2)	0.83 (14)	0.42
Years of school attendance	≥12 <12	5 (62.5) 3 (37.5)	5 (62.5) 3 (37.5)		
Single-parent raising		0 (0)	0 (0)	0	-
Current partnership	yes no	6 (75.0) 2 (25.0)	8 (100.0) 0 (0)	2.29 (1)	0.13
Number of children	1 2 3 4	0 (0) 6 (75) 1 (12.5) 1 (12.5)	1(12.5) 2 (25.0) 4 (50.0) 1 (12.5)	4.8 (3)	0.19
Children					
Age [y]		14.3 (1.9)	15.1 (2.5)	−0.79 (14)	0.44
Sex	Female Male	5 (62.5) 3 (37.5)	7 (87.5) 1 (12.5)	1.33 (1)	0.25
Treatment setting	Inpatient Daily-care	7 (87.5) 1 (12.5)	7 (87.5) 1 (12.5)	0.00 (1) 0.00 (1)	1.00 1.00

Notes: M: mean value, SD: standard deviation, t: test size of paired *t*-test, df: degree of freedom, χ^2^: test size of Chi-square test.

**Table 2:** Father and offspring ratings on internalizing and externalizing problems at hospital admission.

	In-Person Cohort		Online Cohort				
	M (SD)	T ≥ 64	M (SD)	T ≥ 64	t (df)	|d|	*p*
		**n** **(%)**		**n** **(%)**			
Father rating		*n* = 5		*n* = 4			
Internalizing	66.60 (10.02)	3 (60)	70.00 (7.35)	3 (75)	−0.57 (7)	0.40	0.590
Externalizing	57.80 (11.03)	1 (20)	53.25 (6.40)	0 (0)	0.73 (7)	0.50	0.491
Total Problems	63.60 (13.16)	2 (40)	62.00 (6.33)	2 (50)	0.22 (7)	0.16	0.831
Offspring rating		*n* = 8		*n* = 5			
Internalizing	71.88 (3.83)	8 (100)	78.60 (12.46)	5 (100)	−1.17 (11)	0.79	0.300
Externalizing	50.13 (10.11)	0 (0)	51.00 (2.55)	0 (0)	−0.19 (11)	0.11	0.855
Total Problems	65.00 (4.31)	6 (75)	66.20 (8.08)	3 (60)	−0.35 (11)	0.21	0.731

Notes: CBCL/6-18R: Child Behavior Check List [47], YSR: Youth Self-Report [48], T ≥ 64: clinical relevance, M: mean value, SD: standard deviation, t: test size of *t*-test, df: degree of freedom of *t*-test, |d|: Cohen’s d (*t*-test effect size).

**Table 3:** Pre- and post-intervention paternal stress, self-efficacy, and competence.

	Pre-Intervention (t1)	Post-Intervention (t2)			
	M (SD)	M (SD)	t (df)	|d|	*p*
In-person cohort Paternal					
Stress child-related	52.63 (20.47)	50.25 (18.34)	2.01 (7)	0.71	0.042
Stress parent-related	77.13 (25.81)	72.88 (27.59)	1.36 (7)	0.48	0.108
Self-efficacy	23.88 (5.16)	26.63 (4.47)	−2.56	0.57 ^a^	0.006
Competence	55.50 (12.88)	58.50 (14.00)	−1.04 (7)	0.37	0.167
Online cohort Paternal					
Stress child-related	56.00 (10.85)	51.13 (12.12)	1.94 (7)	0.69	0.047
Stress parent-related	67.00 (14.80)	64.00 (16.91)	1.02 (7)	0.36	0.171
Self-efficacy	23.25 (3.77)	25.75 (3.15)	−2.89 (7)	1.02	0.012
Competence	51.00 (11.19)	55.50 (17.23)	−1.41 (7)	0.50	0.101

Notes: n = 8/8 presence/online. PSI: Parenting Stress Inventory [49], FSW: German questionnaire ‘Fragebogen zur Selbstwirksamkeit in der Erziehung’ [50], EBF-KJ: German questionnaire ‘Elternbildfragebogen für Kinder und Jugendliche’ [51], M: mean value, SD: standard deviation, t: test size of *t*-test, df: degree of freedom of *t*-test, |d|: Cohen’s d/*t*-test effect size [For parental self-efficacy, Wilcoxon signed-rank test was performed instead of *t*-test. ^a^ z for the effect size of Wilcoxon signed-rank test)].

## Data Availability

The datasets generated during and/or analyzed during the current study are available from the corresponding author on reasonable request.

## Supplementary Materials

The following supporting information can be downloaded at: https://www.mdpi.com/article/10.3390/bs14010013/s1, Table S1: Pre-(t1) intervention correlations between paternal stress (PSI), self-efficacy (FSW) and competence (EBF-KJ) with child characteristics and sociodemographic factors; Table S2: Pre-(t1) and post-(t2)-intervention correlations between paternal stress (PSI), self-efficacy (FSW) and competence (EBF-KJ).
