# Supplementary material for: The Father’s Part: A Pilot Evaluation of a Father-Centered Family Intervention Group in Child and Adolescent Psychiatry
Source: Behav Sci (Basel). 2023 Dec 22;14(1):13. doi: 10.3390/bs14010013 (PMC10812738; doi:10.3390/bs14010013)
Supplement: Supplementary file 1 [file behavsci-14-00013-s001.zip › behavsci-2745721-supplementary.pdf]

The Father's Part: A pilot Evaluation of a Father-Centered Family Intervention Group in Child and Adolescent Psychiatry

Supplementary Material

For detection of inter-outcome associations and outcome correlations with sociodemographic/child characteristics mediators Pearson's (r_p), Spearman's (r_s), or point-biserial (r_{pb}) correlations were calculated depending on scale levels while $|r| \geq 0.10$ are considered low correlation, $|r| \geq 0.30$ moderate, and $|r| \geq 0.50$ strong/high correlation.

Child age, father partnership status or father years of school attendance were not associated with pre-intervention parenting measures (Table S2). Higher paternal PSE was associated with female adolescents in the in-person cohort ($r(8) = -0.78$, $p = 0.022$). For the online intervention group, higher parent-related paternal stress was associated with male adolescents ($r(8) = 0.87$, $p = 0.005$) (Table S2).

Table S1 Pre-(t1) intervention correlations between paternal stress (PSI), self-efficacy (FSW) and competence (EBF-KJ) with child characteristics and sociodemographic factors.

Paternal…	Stress child-related		Stress parent-related		Self-efficacy		Competence	
	P-FG	O-FG	P-FG	O-FG	P-FG	O-FG	P-FG	O-FG
**Child**								
Age	-0.19	-0.01	0.18	0.41	0.02	-0.57	0.02	-0.11
Sex	0.42	0.63+	0.29	**0.87****	**-.078***	-0.13	-0.33	-0.65+
**Father:**								
Partnership	0.10	-[a]	0.20	-[a]	-0.34	-[a]	-0.41	-[a]
Number of children	0.04	0.04	-0.14	0.22	0.50	0.16	0.16	-0.47
Years of school attendance	0.21	0.17	0.21	0.09	0.08	0.34	0.07	-0.35

Notes: n = 8/8 in-person/online. *P-FG: in-person cohort of fathers' group, O-FG: online cohort of fathers' group,* PSI: Parenting Stress Inventory, *FSW:* German questionnaire 'Fragebogen zur Selbstwirksamkeit in der Erziehung' ,*EBF-KJ*: German questionnaire 'Elternbildfragebogen für Kinder und Jugendliche, [a] partnership constant, ** p < 0.01, * p < 0.05, + p < 0.10.

In the in-person cohort, child-related paternal stress significantly correlated with parent-related paternal stress before and after the father-group intervention (t1: $r(8) = 0.80$, $p = 0.016$; t2: $r(8) = 0.75$, $p = 0.033$; Table S1). There was also a significant negative correlation of child-related paternal stress and paternal PSE at both points of data acquisition (t1: $r(8) = -0.77$ $p = 0.025$; t2: $r(8) = -0.80$, $p = 0.017$). In the online cohort, high child-related paternal stress was associated with low paternal competence at t1 ($r(8) = -0.74$, $p = 0.038$).

Table S2 Pre- (t1) and post-(t2)-intervention correlations between paternal stress (PSI), self-efficacy (FSW) and competence (EBF-KJ).

	In-Person Cohort			Online Cohort		
	1.	2.	3.	1.	2.	3.
**Pre-intervention (t1)**						
**Paternal…**						
1. Stress child-related	–	–	–	–	–	–
2. Stress parent-related	**0.80***	–	–	0.64+	–	–
3. Self-efficacy	**-0.77***	-0.70+	–	-0.26	-0.51	–
4. Competence	-0.51	-0.64+	0.59	**-0.74***	-0.56	-0.16
**Post-intervention (t2)**						
**Paternal…**						
1. Stress child-related	–	–	–	–	–	–
2. Stress parent-related	**0.75***	–	–	0.28	–	–
3. Self-efficacy	**-0.80***	-0.56	–	-0.03	-0.18	–
4. Competence	-0.47	-0.39	0.30	-0.51	-0.27	-0.31

Notes: n = 8/8 in-person/online. PSI: Parenting Stress Inventory , *FSW:* German questionnaire 'Fragebogen zur Selbstwirksamkeit in der Erziehung', *EBF-KJ*: German questionnaire 'Elternbildfragebogen für Kinder und Jugendliche, * p < 0.05, + p < 0.10.
